# Correlation between immune signature and high‐density lipoprotein cholesterol level in stage II/III colorectal cancer

**DOI:** 10.1002/cam4.1987

**Published:** 2019-02-07

**Authors:** Yun Wang, Xiao‐qiang Sun, Hao‐cheng Lin, De‐shen Wang, Zhi‐qiang Wang, Qiong Shao, Feng‐hua Wang, Shu‐mei Yan, Jie‐ying Liang, Zhao‐lei Zeng, Huai‐qiang Ju, Rui‐hua Xu, Yu‐hong Li

**Affiliations:** ^1^ Sate key Laboratory of Oncology in South China, Collaborative Innovation Center for Cancer Medicine Sun Yat‐sen University Cancer Center Guangzhou P.R. China; ^2^ Department of Hematologic Oncology Sun Yat‐sen University Cancer Center Guangzhou P.R. China; ^3^ Department of Medical Oncology Sun Yat‐sen University Cancer Center Guangzhou P.R. China; ^4^ Key Laboratory of Tropical Disease Control Chinese Ministry of Education Zhong‐shan School of Medicine Sun Yat‐sen University Guangzhou P.R. China; ^5^ Department of Molecular Diagnostics Sun Yat‐sen University Cancer Center Guangzhou P.R. China; ^6^ Department of Pathology Sun Yat‐sen University Cancer Center Guangzhou P.R. China

**Keywords:** colorectal cancer, HDL‐C, immune signature

## Abstract

An increasing amount of evidence suggests that high‐density lipoprotein cholesterol (HDL‐C) is related to a positive prognosis in various cancers. However, the correlation between HDL‐C and the immune signature and the prognostic role of HDL‐C in stage II/III colorectal cancer (CRC) has not been previously reported. A total of 667 CRC patients were enrolled and divided into two groups based on the lower limit of normal HDL‐C values (0.78 mmol/L). We used Kaplan‐Meier curves and the Cox regression model to analyze the prognostic role of HDL in both disease‐free survival (DFS) and overall survival (OS). Fifty‐five pairs of tumor tissues were selected according to the variation in HDL‐C levels (high or low) and the matched characterizes (ages, T stage, and N stage). Using immunohistochemistry, tumor tissues were stained with antibodies against CD3, CD8, CD163, iNOS, Forkhead box P3 (FOXP3), and CD33. We calculated the density of positively‐stained infiltrating cells in the tumor center (TC) and invasive margin (IM). We then used Spearman rank correlation to further investigate the relationship between HDL‐C levels and the immune signatures. Our results revealed that compared to patients with high HDL‐C levels, patients with low HDL‐C levels had poor 3‐year DFS (68.9% vs 83.1%, *P* = 0.032) and 5‐year OS rates (66.6% vs 85.3%, *P = *0.002). We also identified a positive correlation between HDL‐C and CD3^+^, CD8^+^ and iNOS^+^ cells and a negative correlation between HDL‐C and CD163^+^ cells in both the TC and IM. This study reveals that a low HDL‐C level in stage II/III CRC patients predicts poor prognosis. The correlation between the HDL‐C level and immune signature in tissue specimens suggested that HDL‐C is likely to play an inhibitory role in tumor development via affecting immune responses.

## 1 INTRODUCTION

Colorectal cancer (CRC) is one of the most prevalent and deadliest types of cancer worldwide. In China, the incidence rate of CRC increases 4%‐6% each year.^1^, ^2^ Although most early‐stage patients receive curative resection survive, the five‐year post‐metastasis survival rate remains approximately 20%‐45%.^3^, ^4^ Cancer metastasis is a complex process with multiple underlying mechanisms. Clarifying both the underlying mechanisms and the potential risk factors of metastasis is imperative for better prevention and treatment of CRC recurrence.

High‐density lipoprotein cholesterol (HDL‐C) was first known for its atheroprotective role, and then, clinical observational studies suggested a protective role of HDL‐C in cancer. A large meta‐analysis of randomized controlled trials indicated that for every 10 mg/dL increase in the plasma HDL‐C level, the risk of cancer incidence is reduced by 36%.^5^ In addition, in numerous cancer cases, the HDL‐C level has been observed to be positively associated with the overall survival rate.^5^, ^6^, ^7^, ^8^ Apolipoprotein A‐I (ApoA‐I),^9^, ^10^, ^11^ the predominant protein in HDL‐C, has also been observed to be positively related to survival. In CRC patients, the level of HDL‐C was significantly lower compared to that in the health controls.^12^ CRC patients with metastases had substantially higher levels of low‐density lipoprotein cholesterol (LDL‐C) and LDL‐C to HDL‐C ratio than early‐stage patients.^13^ Similarly, a Chinese cohort with metastatic CRC also verified the independent prognostic role of high LDL‐C/HDL‐C ratio for poor survival.^14^

Nevertheless, the molecular mechanism underlying apoA‐I/HDL anti‐tumor activity is still unknown. In one study on nasopharyngeal carcinoma, HDL was reported to promote the invasion and migration of nasopharyngeal cancer cells.^15^ Our preliminary in vitro data also showed that HDL‐C had no direct inhibitory effect on the proliferation, migration, and invasion of RKO and HCT116 colon cancer cells. Nevertheless, previous animal studies have supported an anti‐neoplastic role for apoA‐I/HDL‐C. Subcutaneous injections of apoA‐I/HDL‐C mimetic peptides inhibited tumor development in multiple mouse models including ovarian and colon cancer.^16^, ^17^ Moreover, these anti‐tumor effects of apoA‐I/HDL‐C require an immunocompetent host. In these hosts, HDL‐C might affect immune cells in the tumor microenvironment instead of directly killing tumor cells.^18^ In studies comparing syngeneic B16F10L tumors from mice that were either apoA‐I deficient or mice expressing human apoA‐I, the results showed that the increasing levels of apoA‐I/HDL decreased the recruitment of myeloid‐derived suppressor cells (MDSC) and promoted the accumulation of tumor‐associated macrophages (TAMs) with an M1‐like phenotype, and inhibited the accumulation of M2‐like TAMs within tumor beds.^18^, ^19^

However, evidence for the anti‐tumor immune regulatory role of HDL‐C in humans has never been reported. We designed this study to (a) evaluate the prognostic ability of HDL‐C in stage II‐III CRC patients and (b) to compare differences in the immune signature of CRC patients with various HDL‐C levels.

## 2 MATERIALS AND METHODS

### 2.1 Patients

In this study, patients with stage II/III CRC who had undergone curative resection between June 2002 and December 2012 at the Sun Yat‐sen University Cancer Center, China, were analyzed retrospectively. The criteria for eligible patients included the following: pathologically diagnosed stage II/III CRC; history of curative resection, with fluoropyrimidine‐based adjuvant chemotherapy; hepatic function within normal limits (ALT <80 U/L; AST <80 U/L); and the availability of complete clinicopathological information for analysis. For stage II patients, only those who have high‐risk factors and treated with postoperative chemotherapy were included. This study was approved by the institutional ethical review board of Sun Yat‐sen University Cancer Center and was conducted in accordance with the Declaration of Helsinki of the World Medical Association.

### 2.2 Information retrieval and follow‐up

Clinicopathological characteristics were reviewed and collected retrospectively from the patient medical charts. Body mass index (BMI) was recalculated according to the following formula: BMI = weight/height^2^ (in kilograms/meters^2^). Considering the different criteria of lymph node metastasis between the sixth and seventh TNM stage system, the histological tumor samples were reviewed, and the TNM stage was reclassified according to the Union International Control Cancer (UICC) staging system, version 7.^20^ We detected the levels of HDL‐C and other lipids/lipoproteins (including cholesterol, triglycerides, LDL‐C, apoA‐I, and apolipoprotein B, apoB) in pretreatment serum from fasting blood samples. These samples were immediately sent and analyzed by a Hitachi 7600‐020 automatic biochemical analyzer (Hitachi High‐Technologies, Tokyo, Japan). Later, these data were retrieved and analyzed retrospectively. Since the normal range of HDL‐C is 0.78‐1.16 mmol/L, patients with HDL‐C levels less than the lower limit of the normal value of 0.78 mmol/L were defined as having a low HDL‐C level, and those with levels greater than 0.78 mmol/L were defined as having a high HDL‐C level. Postoperative chemotherapy was managed according to the National Comprehensive Cancer Network (NCCN) guideline based on the patient's staging and clinical characteristics. We followed‐up with the patients every 3 months for the first 3 years and every 6 months for the next 5 years. Follow‐up reviews were conducted via reviewing hospital records and making phone calls to the patients or their relatives.

### 2.3 Immunohistochemistry

The corresponding formalin‐fixed paraffin‐embedded tumor tissues were cut into 5‐µm‐thick sections. Immunohistochemistry was performed according to standard indirect immunoperoxidase protocols. The sections were deparaffinized and rehydrated. Endogenous peroxides were quenched, followed by antigen retrieval, which is conducted by incubating with sodium citrate‐hydrochloric acid buffer solution at 95°C for 20 minutes and cooled off by flowing water for 30 minutes. The slides were stained with the following primary antibodies: rabbit anti‐CD3 monoclonal antibody (ZSGS‐BIO; Beijing, China; Catalog Number: ZM‐0417; Dilution: Commercial working solution), rabbit anti‐CD8 monoclonal antibody (ZSGS‐BIO; Catalog Number: ZA‐0508; Dilution: Commercial working solution), rabbit anti‐CD33 monoclonal antibody (ZSGS‐BIO; Catalog Number: ZM‐0045; Dilution: Commercial working solution), rabbit anti‐CD163 monoclonal antibody (ZSGS‐BIO; Catalog Number: ZM‐0428; Dilution: Commercial working solution), rabbit anti‐iNOS monoclonal antibody (Abcam, Cambridge, MA, USA; Catalog Number: ab178945; Dilution: 1:500) and rabbit anti‐FoxP3 monoclonal antibody (Cell Signaling Technology, Boston, MA, USA; Catalog Number: #98377; Dilution: 1:200). All slides were incubated with primary antibodies mentioned above at 4°C overnight. The combination of detection reagent including corresponding secondary antibody and streptavidin‐horseradish peroxidase complex and diaminobenzidine tetrahydrochloride (DAB) (Dako, Santa Clara, CA, USA; Catalog Number: K5007) was then added and incubated under the condition of protection from lights at 37°C for 30 minutes, and the sections were counterstained with hematoxylin.

### 2.4 Quantification of infiltrating immune cells

The staining density of the infiltrating cells in different areas of each tissue section was quantified by two independent physicians with computer‐assistance using ImageJ (National Institute of Health, Bethesda, MD, USA). All tissue samples were analyzed, and the physicians were blinded to the corresponding clinicopathological information. The density of the positive non‐tumor cells was calculated with 20× magnification in five random fields of both the tumor center (TC) and invasive margin (IM). The target region was selected by Polygon Selection of Image J and the area was calculated by the software automatically. Then we counted the positive non‐tumor cells in the correspondent region manually and calculated the densities of positive cell in each field. As iNOS was also expressed in the cytoplasm of colorectal cancer cells, only the positive non‐tumor cells were calculated.^21^ The mean density of the five fields selected by the two physicians, respectively, was used for further analysis. We defined the IM as an area with a 500 µm width on the border between the malignant cells and normal tissue.^22^

### 2.5 Statistical analysis

Kaplan‐Meier curves were used to evaluate the prognostic effect of pretreatment lipid/lipoprotein levels, including those of HDL‐C, LDL‐C, cholesterol, triglycerides, apoA‐I, and apoB, in both disease‐free survival (DFS) and overall survival (OS) after curative resection. DFS was defined as the interval of time between the date of curative surgery and the date of tumor recurrence at any site or the date of death due to any cause; patients who were lost during follow‐up or still alive or disease‐free at the last follow‐up were censored. OS was defined as the interval of time between the date of curative surgery and the date of death due to any reason; patients who were lost during follow‐up or still alive at the last follow‐up were censored. Univariate and multivariate Cox regression analysis were both used to assess the influence of clinicopathological factors on survival. The age, gender, location of primary tumor, tumor grade, T‐stage, N‐stage, tumor size, pre‐operative carcinoembryonic antigen (CEA), pre‐operative CA19‐9, BMI, and pretreatment lipids were first assessed in univariate analysis. Only statistically significant variables (*P* < 0.05) in the univariate analysis were tested in the multivariate analyses by a forward stepwise Cox regression modelling. The chi‐square tests were performed to evaluate the association between HDL‐C and clinicopathological variables of interest. An internal validation set generated by randomly selecting 80% of the original dataset using SPSS software was conducted to verify the results of univariate and multivariate cox analyses. The relationship between the pretreatment HDL‐C level and immune signature indicators mentioned above was then investigated by Spearman rank correlation tests. The statistical analyses presented in this study were conducted with SPSS software version 22(IBM, Armonk, NY, USA) using two‐tailed tests. A value of *P* < 0.05 indicated that the difference between the groups was statistically significant.

## 3 RESULTS

### 3.1 Patient characteristics

Fifty‐five patients were assigned to the low HDL‐C level group, and 612 patients were assigned to the high HDL‐C level group. The baseline characteristics of the 667 total patients are shown in Table 1. According to the table, patient gender was predominately male in both the groups (45 patients and 372 patients in the low and high group, respectively). However, male patients were a greater proportion of the high HDL‐C level group (81.8% vs 60.8%, *P* = 0.002). In both groups, the majority of patients (67.3%, 37 patients in low HDL group and 80.1%, 490 patients in high HDL group, respectively) presented with a normal CA199 level (lower than 30 U/mL), but this proportion was smaller in the low HDL group (*P* = 0.026). In addition, the distribution of BMI status, TNM stage, histological subtypes, preoperative CEA levels, and underlying diseases including diabetes and hypertension were not different between the two HDL‐C groups.

**Table 1:** Clinicopathological characteristics (N = 667)

Characteristics	Low HDL	High HDL	*P* value
	N (%)	N (%)	
Age at diagnosis			
Median (range)	51 (34‐70)	55 (23‐75)	
≤65 y	48 (87.3)	518 (84.6)	0.602
>65 y	7 (12.7)	94 (15.3)	
Gender			
Male	45 (81.8)	372 (60.8)	**0.002**
Female	10 (18.2)	240 (39.2)	
BMI			
Median (range)	23.0 (16.8‐27.1)	22.6 (13.5‐34.3)	
<24 kg/m^2^	35 (63.6)	405 (66.2)	0.703
≥24 kg/m^2^	20 (36.4)	207 (33.8)	
Location of primary tumor			
Colon	34 (61.8)	306 (50.0)	0.093
Rectum	21 (38.2)	306 (50.0)	
Histological subtype			
Non‐mucinous	48 (87.3)	561 (91.7)	0.391^a^
Mucinous	7 (12.7)	51 (8.3)	
Tumor grade			
G1‐2	37 (67.3)	482 (78.8)	**0.050**
G3	18 (32.7)	130 (21.2)	
T‐stage			
pT1‐3	10 (18.2)	128 (20.9)	0.632
pT4	45 (81.8)	484 (79.1)	
N‐stage			
pN0	23 (41.8)	263 (43.0)	0.868
pN1‐2	32 (58.2)	349 (57.0)	
Tumor size			
≤4 cm	31 (56.3)	365 (59.6)	0.636
>4 cm	24 (43.6)	247 (40.4)	
Pre‐operative CEA^b^			
Positive	28 (50.9)	352 (57.5)	0.343
Negative	27 (49.1)	260 (42.5)	
Pre‐operative CA199			
≤30 U/mL	37 (67.3)	490 (80.1)	**0.026**
>30 U/mL	18 (32.7)	122 (19.9)	
Smoker			
Yes	18 (32.7)	133 (21.7)	0.062
No	37 (67.3)	479 (78.3)	
Diagnosis of diabetes			
Yes	5 (9.1)	32 (5.2)	0.373^b^
No	50 (90.9)	580 (94.8)	
Diagnosis of hypertension			
Yes	7 (12.7)	88 (14.4)	0.737
No	48 (87.3)	524 (85.6)	

BMI, body mass index; CEA, carcinoembryonic antigen; CA199, Carbohydrate antigen 199.

a With continuity correction.

b The reference value of CEA: nonsmoker ≤2.5 ng/mL, smoker ≤5 ng/mL.

The value showed in bold highlighted that the difference between the correspondent characteristics groups was statistically significant (*P* < 0.05).

### 3.2 Prognostic value of HDL‐C

**Figure 1:**
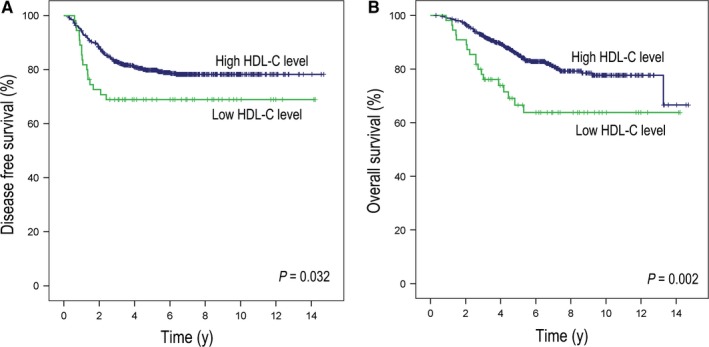
Disease‐free (DFS) and overall survival (OS) between patients with high and low HDL‐C levels. Patients with high HDL‐C levels presented with an improved DFS and OS

In the follow‐up of the datasets, 141 CRC patients experienced disease recurrence and 117 patients died (median follow‐up: 5.5 years [IQR: 3.7‐7.6 years]). Based on the Kaplan‐Meier curves, our results revealed that compared with patients in the high HDL‐C group, patients in the low HDL‐C group presented with a worse DFS (3‐year DFS rate: 68.9% vs 83.1%, *P* = 0.032; Figure 1A) and OS (5‐year OS rate: 66.6% vs 85.3%, *P* = 0.002; Figure 1B). In addition to HDL‐C, other lipids/lipoproteins did not have significant relationship with DFS or OS (See Figure S1 and Table S1). In the Univariate Cox regression analysis, we determined that a low HDL‐C level in patients correlated with not only a poor DFS (hazard ratio: 1.73, 95% CI: 1.04‐2.87, *P* = 0.034; Table 2) but also a poor OS (hazard ratio: 2.18, 95% CI: 1.32‐3.60, *P* = 0.002; Table 2). This difference in DFS and OS was also maintained in the multivariate Cox model (hazard ratio of DFS: 1.73, 95% CI: 1.04‐2.89, *P* = 0.034; hazard ratio of OS: 2.10, 95% CI: 1.26‐3.49, *P* = 0.004; Table 2) and verified in the re‐sample internal validation set (Table S2). Other independent indicators that were associated with an improved DFS and OS rate in both univariate and multivariate Cox analysis included the location of the primary tumor in colon, N stage pN0, and low preoperative CA199 levels. Furthermore, weak independent indicators include advanced T stage for DFS and older age and male gender for OS.

**Table 2:** Predictive factors for survival by univariate and multivariate analysis

		Univariate analysis		Multivariate analysis	
		HR (95%CI)	*P* value	HR (95%CI)	*P* value
Disease‐free survival					
Location of primary tumor	Colon vs rectal	0.68 (0.48‐0.95)	0.023	0.65 (0.46‐0.91)	0.011
T‐stage	pT1‐3 vs pT4	0.53 (0.32‐0.88)	0.015	0.51 (0.31‐0.85)	0.010
N‐stage	pN0 vs pN1‐2	0.39 (0.27‐0.58)	<0.001	0.39 (0.27‐0.58)	<0.001
Pre‐operative CEAa	pos vs neg	1.48 (1.05‐2.10)	0.026		ns
Pre‐operative CA199 (U/mL)	>30 vs ≤ 30	1.62 (1.12‐2.34)	0.010	1.48 (1.02‐2.15)	0.039
HDL‐C level	low vs high	1.73 (1.04‐2.87)	0.034	1.73 (1.04‐2.89)	0.034
Overall survival					
Age (years)	>65 vs ≤ 65	1.79 (1.17‐2.75)	0.008	1.79 (1.16‐2.76)	0.009
Gender	Male vs Female	1.61 (1.07‐2.41)	0.022	1.63 (1.08‐2.46)	0.021
Location of primary tumor	colon vs rectal	0.57 (0.39‐0.82)	0.003	0.53 (0.36‐0.77)	0.001
N‐stage	pN0 vs pN1‐2	0.41 (0.28‐0.62)	<0.001	0.40 (0.26‐0.60)	<0.001
Pre‐operative CEA^a^	pos vs neg	1.50 (1.03‐2.20)	0.037		ns
Pre‐operative CA199 (U/mL)	>30 vs ≤ 30	1.58 (1.06‐2.35)	0.026	1.52 (1.01‐2.29)	0.043
HDL‐C level	low vs high	2.18 (1.32‐3.60)	0.002	2.10 (1.26‐3.49)	0.004

a The reference value of CEA: nonsmoker ≤2.5 ng/mL, smoker ≤5 ng/mL.

### 3.3 HDL‐C‐related immune signature

**Figure 2:**
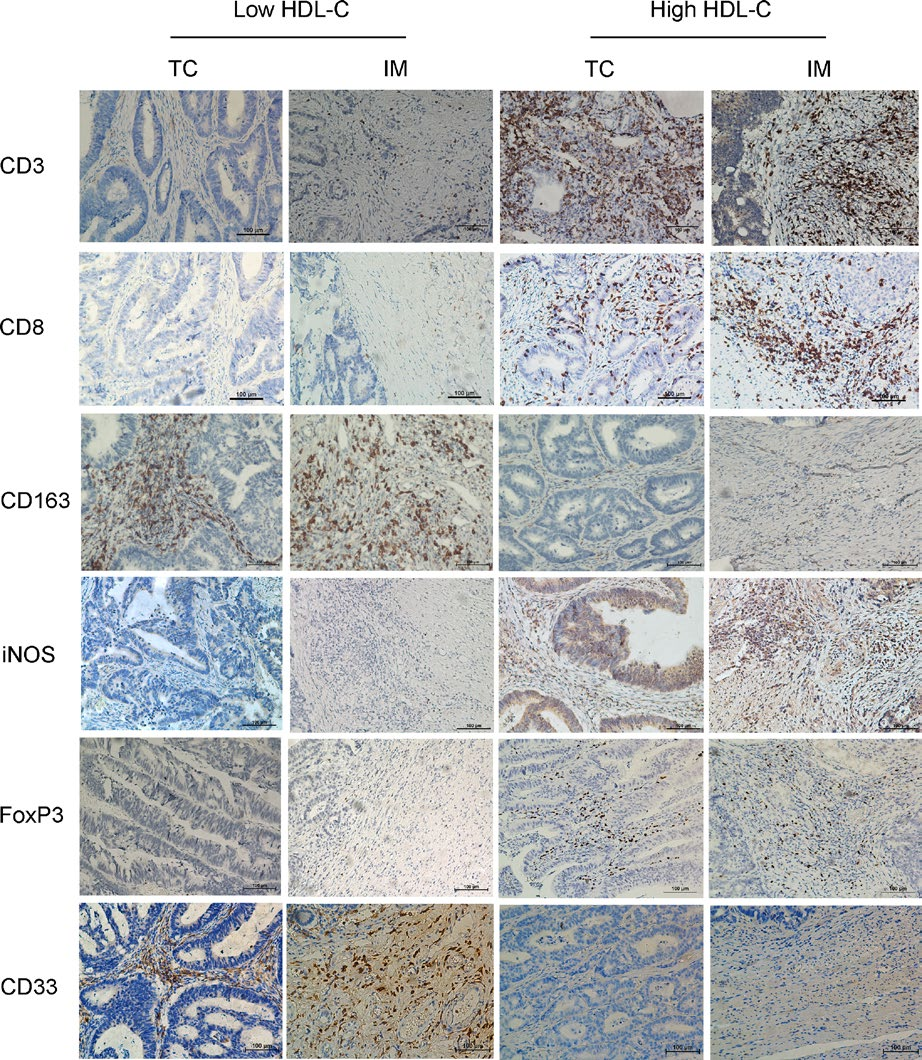
Representative pictures of low‐and high‐density CD3^−^, CD8^−^, CD163^−^, iNOS^−^, FOXP3^−^ and CD33‐positive cells in the tumor center (TC) and invasive margin (IM)

**Figure 3:**
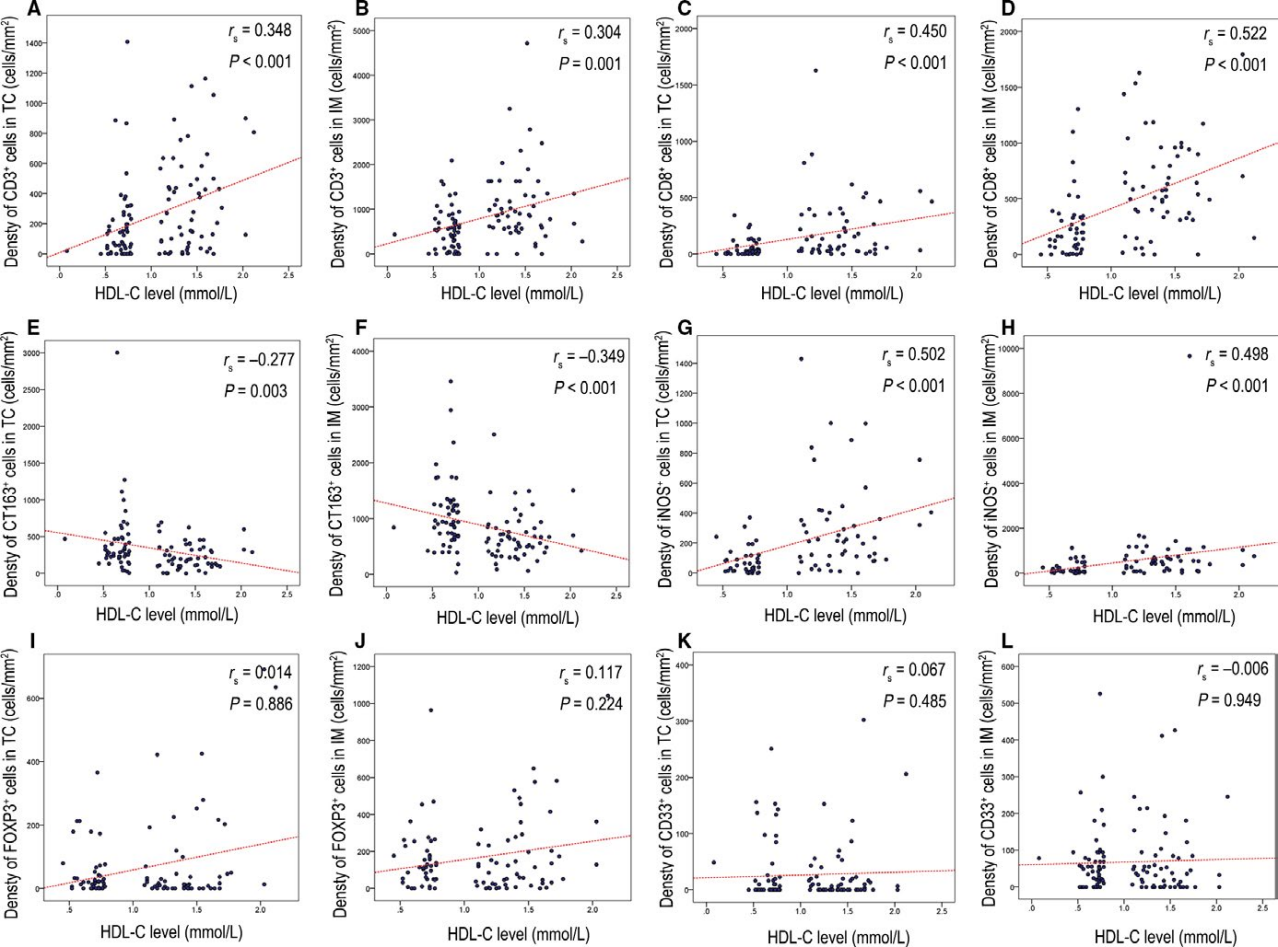
Correlation between HDL level and the immune signature. (A) Analysis of the positive correlation between HDL levels and CD3^+^ cells in the TC; (B) Analysis of the positive correlation between HDL levels and CD3^+^ cells in the IM; (C) Analysis of the positive correlation between HDL levels and CD8^+^ cells in the TC; (D) Analysis of the positive correlation between HDL levels and CD8^+^ cells in the IM; (E) Analysis of the correlation between HDL levels and CD163^+^ cells in the TC; (F) Analysis of the correlation between HDL levels and CD163^+^ cells in the IM; (G) Analysis of the correlation between HDL levels and iNOS^+^ cells in the TC; (H) Analysis of the correlation between HDL levels and iNOS^+^ cells in the IM; (I) Analysis of the correlation between HDL levels and FOXP3^+^ cells in the TC; (J) Analysis of the correlation between HDL levels and FOXP3^+^ cells in the IM; (K) Analysis of the correlation between HDL levels and CD33^+^ cells in the TC; (L) Analysis of the correlation between HDL levels and CD33^+^ cells in the IM

To further explore the relationship between the immune signature and HDL‐C levels, 55 patients with HDL‐C levels exceeding 1.05 mmol/L from the high HDL‐C group were selected by three characteristics, age at diagnosis, T stage and N stage, to match those of the 55 patients in the low HDL group. We then explored the correlation between HDL‐C level and the immune signature, including the staining density of CD3, CD8, CD163, iNOS, Forkhead box P3 (FOXP3), and CD33. The staining density of positive cells in the TC and IM was analyzed. The results revealed that different expression levels of the immune signatures were identified in both the low and high HDL‐C groups (Figure 2). Spearman rank correlation was then used to investigate the relationship between the HDL‐C value and the HDL‐related immune signature (Figure 3). A positive correlation between the HDL‐C level and staining density of CD3^+^ (TC: the Spearman rank correlation coefficient[*r*_s_] = 0.348, *P < *0.001; IM: *r*_s_ = 0.304, *P* = 0.001), CD8^+^ (TC: *r*_s_ = 0.450, *P < *0.001; IM: *r*_s_ = 0.522, *P < *0.001) and iNOS^+^ (TC: *r*_s_ = 0.502, *P < *0.001; IM: *r*_s_ = 0.498, *P* < 0.001) was detected in both the TC and IM. At the same time, a negative correlation between the HDL‐C level and staining density of CD163^+^ cells (TC: *r*_s_ = −0.277, *P* = 0.003; IM: *r*_s_ = −0.349, *P < *0.001) was observed in both the TC and IM. However, the correlation between the HDL‐C level and staining density of CD33^+^ cells (TC: *r*_s_ = 0.067, *P* = 0.485; IM: *r*_s_ = −0.006, *P* = 0.949) and FOXP3^+^ cells (TC: *r*_s_ = 0.014, *P = *0.886; IM: *r*_s_ = 0.117, *P = *0.224) was not statistically significant in either TC or IM.

## 4 DISCUSSION

In this study, we reported that the preoperative HDL‐C level predicted DFS and OS in a cohort of stage II/III Chinese CRC patients after curative resection. A low HDL‐C level indicated poor postoperative survival. Furthermore, the patient's HDL‐C level was associated with numerous immune biomarkers, including CD3, CD8, CD163, and iNOS. To the best of our knowledge, this is the first research that has explored the immune signature of HDL‐C in tissue specimens from CRC patients.

Previous studies have reported that the levels of apoA‐I and HDL‐C are inversely related to not only the risk of colon cancer but also the prevalence of advanced colorectal adenomas.^23^, ^24^ It was reported that every 16.6 mg/dL increase in the HDL‐C level leads to a 22% decrease in colon cancer risk, a 12% increase in non‐advanced colorectal adenoma risk and a 16% decrease in advanced colorectal adenoma risk.^23^ Furthermore, in this study, our results revealed the correlation between HDL‐C level and CRC patients’ prognosis after curative resection. This finding suggests the role of HDL‐C in the progression of CRC metastases. However, the explicit mechanism of apoA‐I/HDL‐C anti‐tumor activity remains indistinct.

The subtle differences in the composition of infiltrating immunocytes under different microenvironment may determine both the prognosis and treatment response in CRC. Prior in vivo studies suggested that the overall net impact of host apoA‐I/HDL‐C levels on the tumor microenvironment is profound and manifold.^16^, ^18^, ^19^ We further made comparison between the differences in the immune signature of CRC patients with various HDL‐C levels. The data indicate that immune markers including CD3, CD8, CD163, and iNOS are all correlated with the HDL‐C level.

Among these markers, CD3 and CD8 show the strongest correlation with HDL‐C level, which is consistent with a prior study in a murine model, that ApoA‐I/HDL‐C could promote the infiltration of cytotoxic CD8 T cells into the tumors of mice expressing human ApoA‐I.^19^ CD3^+^ cells, indicative of pan‐T‐cell expression, is an integral predictor of improved outcome, and regulated by multiple modulating factors including angiogenesis, homing factors, cytokines, and tumor genotype and neurological signals.^25^ Among them, the CD8^+^ cytotoxic T cells was known to be a prototypical anti‐tumor immune cells for the ability to recognize tumor cells in an antigen‐specific manner and secrete cytotoxic molecules to perform anti‐tumor ability.^25^ The significant positive correlation between CD3 and CD8 with HDL‐C level indicate that the microenvironment of high HDL‐C may facilitate the activity and recruitment of CD3^+^ and CD8^+^ T cells. However, contrary to the phenomenon observed in cancer, HDL‐C was reported to attenuate the inflammatory activity of T cells by promoting the free cholesterol efflux and result in an increased fraction of T regulatory cells (Tregs) which attenuate inflammation.^26^ Further investigation on the regulatory role of HDL‐C on tumor‐infiltrating lymphocytes was still warranted under the tumor microenvironment.

There are several distinct populations of TAM that share features of both M1 and M2 macrophages in the tumor tissue. In most cancers, TAM was generally recognized as an anti‐inflammatory, or M2‐like phenotype. However, in colorectal cancer, a higher frequency of pro‐inflammatory, or M1‐like phenotype macrophages was observed in tumor tissues when compared to non‐tumor bowel.^27^ The M2‐like phenotype of TAM was reported to possess the function of metastasis‐promotion, angiogenesis and immunosuppression, while the M1‐like phenotype macrophages are reported to inhibit the growth of tumor.^28^ Therefore, contrary to other cancers, higher infiltration of macrophages in CRC predicted a superior survival.^29^ In this study, the high HDL‐C level condition was observed to negatively correlate with CD163 staining density, which is a marker of M2‐like phenotype of TAM, but was also positively correlated with iNOS, a marker of M1‐like phenotype. This suggest that HDL‐C is possibly involved in the process of converting the M2‐like to an M1‐like phenotype and may partly explain the improved outcome in high HDL‐C population. It was also in concordance with the previous conversion of TAMs from an M2‐ to an M1‐like phenotype contributing to tumor inhibition in immunocompetent murine tumor models.^18^, ^19^ However, HDL‐C was more likely to directly promote the conversion of macrophage cells from a pro‐inflammatory (M1‐like) into an anti‐inflammatory (M2‐like) phenotype in the vitro culture.^30^ The complex tumor microenvironment may account for these conflicting results. Identifying a definite mechanism requires further investigation. And given the heterogeneity and plasticity of macrophage phenotype, analyses combining multiple markers is essential for further identification of macrophage sub‐populations.

The staining density of FOXP3, a crucial marker for Tregs, and CD33, one of tumor promoting MDSC immune markers, was not significantly correlated to HDL‐C level in the human specimens of the current study, although HDL‐C was demonstrated to inhibit the accumulation of MDSC in murine tumor models.^18^, ^19^ However, considering the diversity of CD33 staining immune cells, other immune markers and multiparametric flow cytometry was needed to further confirm the results.

Although our study has revealed the prognostic role of HDL‐C in stage II/III CRC and its immune signature in human tissues, our findings have limitations due to the retrospective design and single‐center data and are unable to further investigate the underlying mechanism of the anti‐tumor ability of HDL‐C. There were 84 real patients of the 667 cases cohort (12.6%) and 2 of the 110 patient cohort (1.8%) who have received preoperative radiotherapy/chemoradiotherapy, which could be a potential confounding factor since chemoradiotherapy could affect the serum HDL‐C levels and tumor immune cell infiltration. Moreover, only high‐risk stage II patients who have received adjuvant treatment was included in this study, which may weaken the generalizability of the results. In this study, predetermined cut‐offs based on the clinical practice were used for analysis, an alternative method such as ROC analysis may reveal more cut‐offs for predicting patient prognosis. In addition to HDL‐C, the levels of LDL‐C, apoA‐I, apoB, cholesterol, and triglycerides were also measured and analyzed in this study. As there were multiple independent hypotheses examining in the same set of data, the offset of multiple hypothesis test should also be considered, although there is a divergence of opinions on multiple hypothesis test. The results with *P* value near 0.05 should be interpreted with caution. In the future, we hope to conduct further multicenter studies and fundamental mechanistic studies on the prognostic role of HDL‐C.

## 5 CONCLUSION

Our research has revealed that patients with preoperative low HDL‐C levels present with poor DFS and OS. Positive correlations were observed between HDL‐C and CD3^+^, CD8^+^ and iNOS^+^ cells. Negative correlations were discovered between the HDL‐C level and CD163^+^ cells in both the TC and IM. Our research suggested that a high HDL‐C level is likely to play an inhibitory role in tumor development via affecting immune responses. However, the mechanism underlying the anti‐tumor activity of HDL‐C still needs further clarification.

## INFORMED CONSENT

Informed consent was obtained from all individual participants included in the study.

## ETHICAL APPROVAL

All procedures performed in this study involving human participants were in accordance with the ethical standards of the institutional research committee and the 1964 Helsinki Declaration and its later amendments or comparable ethical standards.

## CONFLICT OF INTEREST

None declared.

## Supporting information

FigS1
