# Supplementary figures and images for: Correlation between immune signature and high‐density lipoprotein cholesterol level in stage II/III colorectal cancer
Source: Cancer Med. 2019 Feb 7;8(3):1209–17. doi: 10.1002/cam4.1987 (PMC6434197; doi:10.1002/cam4.1987)

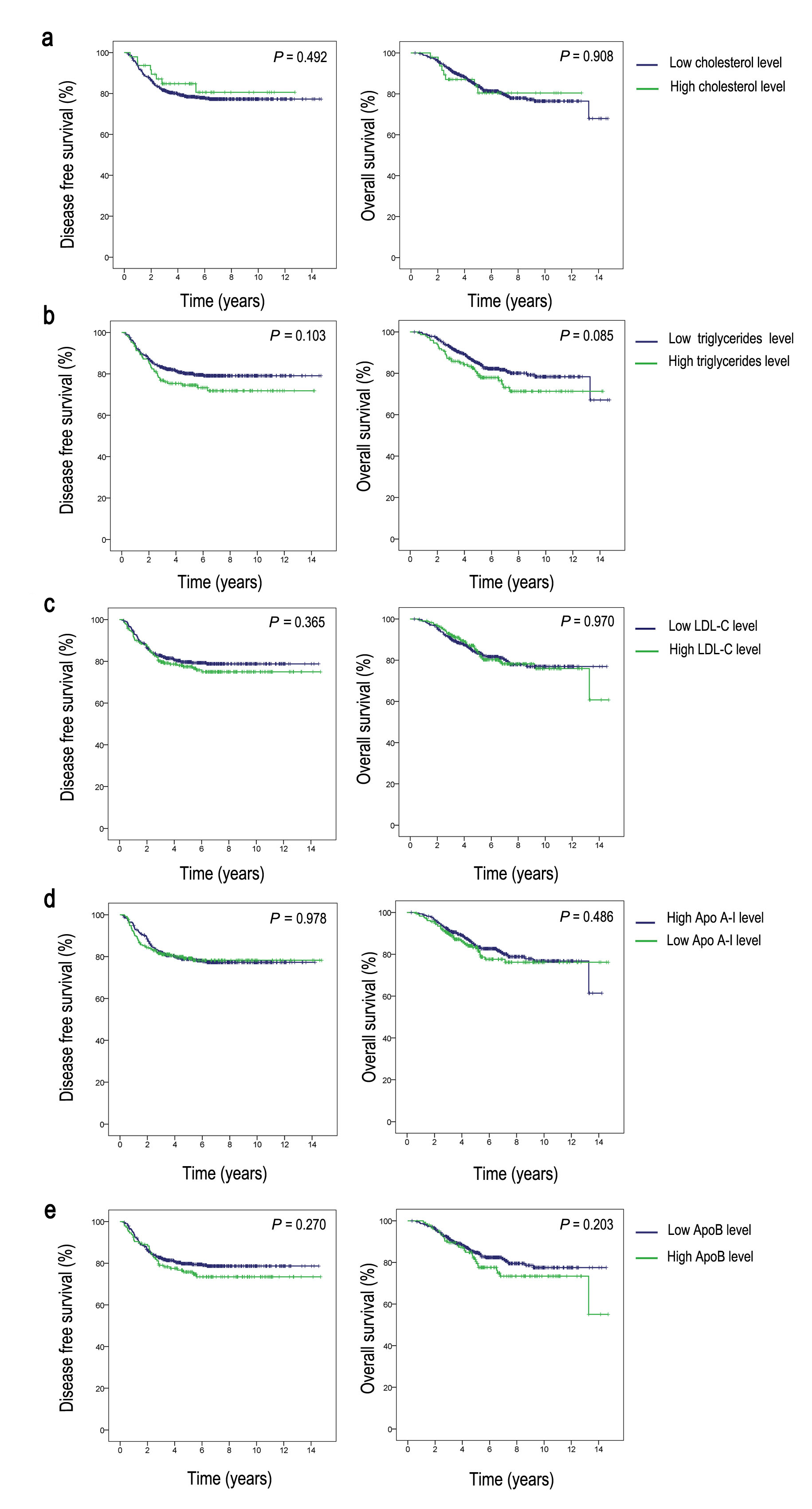

Supplement: Supplementary file 1 — FigS1 [file CAM4-8-1209-s001.tif]
