# Supplementary material for: Correlation between immune signature and high‐density lipoprotein cholesterol level in stage II/III colorectal cancer
Source: Cancer Med. 2019 Feb 7;8(3):1209–17. doi: 10.1002/cam4.1987 (PMC6434197; doi:10.1002/cam4.1987)
Supplement: Supplementary file 2 [file CAM4-8-1209-s002.docx]

**Supplementary Table 1:** The comparison of survival rates including 3-year DFS and 5-year OS rates between patients with low and high levels of lipids/lipoproteins including cholesterol, LDL, ApoA-I, Apo B and triglycerides.

|  | Low | High | *P* values |
| --- | --- | --- | --- |
| Cholesterol^*^ |  |  |  |
| 3-year DFS rates | 81.7% | 84.8% | 0.492 |
| 5-year OS rates | 84.0% | 80.5% | 0.908 |
| LDL-C^†^ |  |  |  |
| 3-year DFS rates | 82.8% | 80.2% | 0.365 |
| 5-year OS rates | 83.6% | 83.8% | 0.970 |
| ApoA-I^‡^ |  |  |  |
| 3-year DFS rates | 81.6% | 82.1% | 0.978 |
| 5-year OS rates | 83.3% | 84.0% | 0.486 |
| ApoB^§^ |  |  |  |
| 3-year DFS rates | 82.7% | 79.0% | 0.270 |
| 5-year OS rates | 84.7% | 80.4% | 0.203 |
| Triglycerides^**^ |  |  |  |
| 3-year DFS rates | 83.4% | 76.9% | 0.103 |
| 5-year OS rates | 85.1% | 75.9% | 0.085 |

* Cutoffed by the high limit of normal: 5.69mmol/L.

† LDL-C: Low-density lipoprotein cholesterol. Cutoffed by the high limit of normal: 3.10mmol/L

‡ Cutoffed by the low limit of normal: 1.20g/L.

§ Cutoffed by the high limit of normal:1.10g/L.

** Cutoffed by the high limit of normal: 1.70mmol/L.

|  |  | Univariate analysis | |  | Multivariate analysis | |
| --- | --- | --- | --- | --- | --- | --- |
| **Disease-free survival** |  | HR (95%CI) | *P* value |  | HR (95%CI) | *P* value |
| Location of primary tumor | colon *vs*. rectal | 0.69 (0.47-0.99) | **0.044** |  | 0.64 (0.44-0.92) | **0.017** |
| N-stage | pN0 *vs*. pN1-2 | 0.45 (0.30-0.68) | **< 0.001** |  | 0.47 (0.31-0.71) | **< 0.001** |
| pre-operative CA199 (U/ml) | > 30 *vs*. ≤ 30 | 1.73 (1.16-2.58) | **0.007** |  | 1.66 (1.11-2.48) | **0.014** |
| HDL-C level | low *vs*. high | 1.78 (1.03-3.06) | **0.038** |  | 1.83 (1.06-3.15) | **0.030** |
| **Overall survival** |  |  |  |  |  |  |
| Age (years) | > 65 *vs*. ≤ 65 | 1.64 (1.03-2.62) | **0.039** |  |  | **ns** |
| Gender | Male *vs.* Female | 1.58 (1.01-2.46) | **0.044** |  | 1.73 (1.10-2.71) | **0.018** |
| Location of primary tumor | colon *vs.* rectal | 0.59 (0.39-0.88) | **0.011** |  | 0.54 (0.36-0.82) | **0.004** |
| N-stage | pN0 *vs.* pN1-2 | 0.45 (0.29-0.71) | **0.001** |  | 0.44 (0.28-0.69) | **< 0.001** |
| pre-operative CA199 (U/ml) | > 30 *vs.* ≤ 30 | 1.82 (1.19-2.80) | **0.006** |  | 1.77 (1.15-2.73) | **0.009** |
| HDL-C level | low *vs.* high | 2.30 (1.35-3.94) | **0.002** |  | 2.28 (1.32-3.91) | **0.003** |

**Supplementary table2：Univariate and multivariate analysis in internal validation set.**

a: The reference value of CEA: nonsmoker ≤ 2.5ng/ml, smoker ≤ 5ng/ml.
